# A randomized, unblinded, controlled clinical study to assess the mobile digital health application INKA in the management of therapy refractory overactive bladder and mixed incontinence

**DOI:** 10.3389/fdgth.2026.1610663

**Published:** 2026-04-10

**Authors:** L. Schramm, A. Wiedemann, C. Neumeister, H. Wolf, Marcus Rudolf Götz, Ch. Neubauer, A. Hegele, R. Eckert, L. Najjari, J. Salem, T. H. Kuru

**Affiliations:** 1 Department of General Psychology and Methodology, University of Bamberg, Bamberg, Bavaria, Germany; 2 Medical Science Department, Dr. Pfleger Arzneimittel GmbH, Bamberg, Germany; 3 Department of Urology, Evangelic Hospital of Witten, Witten, Germany; 4 Department of Geriatrics, University of Witten/Herdecke, Witten, Germany; 5 Research and Development Department, Bode Chemie GmbH, Hamburg, Germany; 6 Urological Center Mittelhessen, Biedenkopf, Germany; 7 Department of Urology, Medical School, University Hospital Marburg, Marburg, Germany; 8 Urologicum Dr. med. Ralf Eckert, Lutherstadt Eisleben, Germany; 9 Department of Gynaecology and Obstretrics, University Clinic RWTH Aachen, Aachen, Germany; 10 Department of Urology, Faculty of Health Sciences Brandenburg, Brandenburg Medical School Theodor Fontane, Brandenburg an der Havel, Germany; 11 CUROS Urology Center, Cologne, Germany

**Keywords:** DIGA, DTx (digital therapeutics), mHealth (mobile health), digital self-management, overactive bladder (OAB), urinary incontinence, bladder training, pelvic floor muscle training

## Abstract

**Background:**

This exploratory, two-arm, randomized, unblinded, controlled, multicentre study assessed the health benefits of the INKA app, a MDR class I CE-marked digital therapy companion for patients with overactive bladder (OAB) and mixed incontinence (MI). INKA offers self-guided educational, behavioural, and motivational content, along with physiotherapy modules and supports daily self-management, in accordance with current clinical guidelines.

**Methods:**

251 patients under first-line stable pharmacological treatment were recruited at 35 study sites in Germany and randomized to receive access to the INKA app or standard of care alone (the control group). Self-assessed OAB related endpoints were investigated at baseline, after 4 and after 12 weeks. The end-of-study visit was conducted on site.

**Results:**

Among 111 evaluable patients (43 INKA, 68 control), baseline characteristics were comparable (mean age 52.7 years, SD 14.6; 27% male, 73% female). 55% of INKA users engaged with the app on a daily basis. At 12 weeks, the INKA group showed a mean reduction of −1.02 (SD 3.36) micturitions per 24 h compared to +0.08 (SD 2.97) in the control group. Significant and clinically meaningful improvements were observed in female INKA users and those with heightened symptom severity. A significant mean increase in urine volume per micturition was noted in the INKA group (+15.75 mL, SD 49.74) vs. the control group (−8.84 mL, SD 52.14), in “OAB wet” and in the female subgroup. The ICIQ-OAB questionnaire results indicated favourable outcomes for all groups, with all INKA patients and the female subgroup showing clinically relevant symptom relief. Additionally, greater improvement on the ICIQ-OABqol questionnaire was reported for the INKA group (−12.5, SD 20.17) vs. the control group (−7.89, SD 20.15). No INKA-related adverse events or device deficiencies were reported.

**Conclusion:**

This proof-of-concept study highlights the potential of the INKA mobile app to reduce micturition frequency and increase the micturition volume in therapy refractory OAB patients, both recognized as key factors of OAB symptom burden. A forthcoming trial will evaluate an optimized and more user-friendly version of the app with patients with a higher symptom severity at baseline.

**Clinical Trial Registration:**

German Clinical Trials Register (DRKS ID 00029329).

## Introduction

Overactive bladder (OAB), also known as urge or urge-frequency syndrome, is a common chronical urological condition characterized by urgency with or without incontinence, frequency and nocturia (1). OAB prevalence is approximately 16% in Europe and the USA, with increasing rates with age, notably after 44 years in women and 64 years in men (2, 3).

Clinical assessment of OAB is based on history-taking by symptom-based questionnaires. Physical examination and diagnostic investigations, such as urinalysis, aim to rule out other potential causes of symptoms (4). OAB significantly impacts the physical, social, and emotional well-being, contributing to anxiety, depression, and embarrassment due to chronicity of symptoms and fear of urinary incontinence (UI) (5). Unfortunately, many individuals refrain from seeking medical assistance, either due to embarrassment or wrongly attributing symptoms to the natural aging process (6).

International medical guidelines prioritize conservative treatments as the initial management strategy for OAB (4, 7, 8). If symptoms persist, pharmacological approaches such as antimuscarinics and beta-3 adrenergic receptor agonists are considered despite their known contraindications and side effects (4, 9). Another option is the transurethral application of botulinumtoxin-A in the detrusor muscle with good outcome. Nevertheless, the procedure is invasive and positive effects remain for 8 months (10, 11). Therefore, therapeutic options remain insufficient and new approaches with good efficacy profiles and minimal side effects are urgently needed (12).

Digital health applications (referred to as DiGAs in German) utilize digital technologies to empower and treat patients (13). These CE-marked medical devices fall into lower risk classes I and IIa according to the European Medical Device Regulation (MDR). To be approved by the German regulatory authority, become eligible for prescription by doctors and psychotherapists, as well as be reimbursed by statutory health insurance funds, DiGAs must adhere to rigorous requirements related to data protection, quality, interoperability, information security, and accessibility. Additionally, they must provide scientific evidence of health benefits for patients through clinical studies (14, 15).

The DiGA candidate INKA integrates multiple functionalities for management of OAB by patients, including educational sources, regular documentation of medication adherence, tracking of fluid intake and caffeine consumption, as well as guidance on pelvic floor exercises and bladder training. With these features, the app systematically monitors therapy progression, captures specific symptoms and behavioural modifications, and provides patients with a simplified overview of their improved outcomes, thereby encouraging patient motivation and compliance. Furthermore, by facilitating direct transmission of data to attending healthcare providers, INKA streamlines the management of OAB, offering a comprehensive solution for both patients and clinicians.

The aim of this clinical trial was to provide evidence for the positive healthcare effects of a complementary therapy with INKA for OAB patients with or without UI, or in combination with stress UI (mixed incontinence), in addition to stable pharmacological treatment.

## Methods

### Intervention

The digital health app INKA (software version 1.0) was developed by DataArt GmbH, Munich, for use on Android and iOS devices. INKA is classified as a medical device software under the EU Medical Device Regulation (MDR) as risk class 1. The app content is based on the latest treatment guidelines for OAB with or without UI (8). Conformity with the MDR and CE-marking was obtained through clinical validation using equivalent devices and software validation of clinically validated questionnaires in accordance with MDCG 2020-1 (Guidance on clinical evaluation (MDR)/Performance evaluation (IVDR) of medical device software).

The self-guided INKA app offers evidence-based education on OAB, covering its causes, symptoms, and associated behaviours such as drinking and caffeine consumption (16, 17). INKA motivates patients to maintain a diary of fluid intake and micturitions, reminds them on medication intake, and provides a graphical evaluation of disease-related parameters to visualize therapy improvement (18). The physiotherapy module, developed in cooperation with a medical expert for physical and rehabilitative medicine, provides text- and video-based guided pelvic floor training, with four phases (learning basic principles, muscle strengthening, endurance training, and strength exercises), automatically adjusted based on training progression (19, 20). INKA further automatically prompts bladder training sessions, which consist of muscle tension exercises and different cognitive distraction games. Bladder training is prompted to delay voiding, according to the reported average numbers of micturition of the previous week and in acute situations (defined as the desire to delay emptying the bladder despite the urge to urinate) via the urgency button on the home screen (21). Collected data can be exported as a pdf file and made available to the physician. The intended daily usage time of the app ranges from 25 to 50 min, with bladder training accounting for the majority of usage time. The recommended usage duration for each participant is automatically individually adjusted based on the in-app reported current symptom status in the ICIQ-UI SF questionnaire. Patients were motivated for daily use through an in-app reward system in the form of collectible stars. Study participants received assistance in case of technical problems. At study initiation, intermittent system failures and downtimes occurred that were limited to system availability and delivery of automated emails and notifications. These were fixed mostly within 3 days and did not affect study methodology.

### Trial design and trial data

This proof-of-concept trial had a national, multicentric, parallel-group, randomised, unblinded, and controlled design and followed applicable ethical and regulatory requirements. Approval of the ethics committee responsible for the coordinating investigator was obtained (University of Witten/Herdecke, June 9th 2022). The study was registered at the German Clinical Trials Register on June 9, 2022 (DRKS ID 00029329). Study synopsis of the final report is publicly accessible (https://www.drks.de/search/de/trial/DRKS00029329/details). The trial is reported in accordance with CONSORT 2025 (Supplementary File 1) and CONSORT-EHEALTH (Supplementary File 2) (22, 23).

### Patients

Due to specifications of the German Health Authority, eligible adult patients were diagnosed with OAB with or without UI and suffered from persisting OAB symptoms despite receiving stable pharmacological treatment for at least four weeks prior to screening. Eligible patients were required to have sufficient German language skills and access to, and proficiency in using a smartphone. Patients were excluded from the clinical investigation if they had other urinary tract conditions (e.g., malignancy or neurological disease), visual blood in urine, three or more urinary tract infections during the last 12 months or current botulinum toxin treatment of the urinary bladder. Written informed consent was obtained from all patients before being included in the study. According to the responses of the patients to the ICIQ-UI-SF at baseline, investigators assigned patients to the “OAB wet” cohort if they suffered from UI or MI. Patients without incontinence were assigned to the “OAB dry” cohort. Further, subgroups of males, females and females with high symptom severity (defined as 8 or more micturitions per 24 h as reported in the bladder protocol at baseline) were analyzed. Patients received a remuneration for study participation.

### Study outcome

All reported study outcomes were self-assessed. ICIQ questionnaires were used in an electronic version. The primary outcome was the change in the mean number of micturitions per 24 h from baseline to 12 weeks based on the three days ICIQ bladder protocol data (24). Further secondary outcomes derived from the bladder protocol were changes in the mean micturition volume, intensity of urge (bladder sensation code 2 to 4), and the number of urine leakages per 24 h (bladder sensation code 4). In contrast to the ICIQ specifications the number of diurnal (from 6 am to 10 pm) and nocturnal (from 10 pm to 6 am) micturitions were calculated using fixed time frames. The changes in OAB specific symptoms and impact on patients' quality of life, were assessed with the patient reported outcome measures (PROMs) ICIQ-OAB (25, 26), ICIQ-OABqol (27–29), and ICIQ-UI SF (30), and the one-hour pad test (31–33). The ICIQ-OAB evaluates OAB symptoms over the preceding 4 weeks, including 4 items, resulting in a total score ranging from 0 to 16. The ICIQ-OABqoL evaluates QoL in patients with OAB and includes 26 items, resulting in an overall score of 25 to 160. The ICIQ-UI SF evaluates the frequency, severity and impact on QoL of urinary incontinence, including 4 items, yielding a total score from 0 to 21. For all ICIQ instruments a higher score indicates increased burden. Thresholds for MCIDs were a ≥ 15% reduction for the primary parameter and a ≥ 1.0 reduction in the total score for the ICIQ-OAB. The one-hour pad test is a non-invasive test that measures urine loss by weighing the pads before and after physical exercise (based on ICS standardized protocol) (33). In addition, safety data was collected.

### Recruitment and randomization

Study patients were recruited at 35 study sites across Germany (urological practices, clinics). Random allocation sequence was generated by the contract research organization using SAS® 9.4 software. Randomization was stratified by gender using block size four, with a total of 100 blocks. Patients were randomized automatically via the eCRF. Study staff enrolling participants had no access to the sequence prior to assignment. Randomization was performed in a 1:1 ratio in the two cohorts (with urinary incontinence (UI) or without UI) to either the intervention group (INKA app users in addition to standard of care) or the control group (standard of care alone). Neither patients nor investigators were blinded due to the nature of the study.

### Collection and handling of data

Study assessments were performed at baseline, week 4 and week 12. As per regulatory requirement, assessments were collected through the study-specific web portal, and for INKA users also in the INKA app, and directly transferred to the database. During baseline and the end-of-study (EOS) visit, source data was collected at the study sites and transferred to the eCRF. After the EOS, no post-study follow-up was performed. Technical difficulties with the web portal and INKA app resulted in missing or duplicated data for several variables. Beside sensitivity analyses (LOCF), no missing data was imputed. Duplicate data occurred since the app did not confirm a successful entry. Therefore, entries which have obviously been recorded twice within ≤1 min were treated as duplicates and merged to a single entry (<2% of bladder protocol data). Safety data were reported digitally or in person to the study sites. Remote monitoring was performed. Anonymized app user data were not part of the clinical trial database but were evaluated separately.

### Sample size and statistical methods

In accordance with the descriptive nature of the study, no formal power calculation was performed. An initial sample size of 216 patients was chosen to be sufficient in order to get estimates of the documented parameters. Based on the results of the exploratory interim analysis (dated 12th April 2023) calculation of the sample size led to an adjustment to 250 patients. The data presented are the result of the final analyses conducted using the full analysis set (FAS), consisting of all patients with completed baseline assessments and with at least one post baseline assessment regarding the primary outcome. The number of patients in FAS for the different outcome parameters varied according to the availability of these assessments at week 12. Subgroup analyses were conducted in the same manner as for the total cohort. Safety analysis was performed on all randomized patients (Intention-to-treat set, ITT). Results are provided by summary statistics for quantitative data. Two-sided, exploratory statistical tests were performed on a descriptive level and without adjustment for multiplicity, using SAS® 9.4. Sensitivity analyses were performed using the LOCF approach.

## Results

### Recruitment and baseline characteristics

Participants on stable pharmacological treatment for OAB were recruited from July 5th 2022 to April 13th 2023. Randomized participants received a QR code at the study site to access the web portal for study specific assessments (both treatment groups) and, in the intervention group, to download the INKA app to their mobile device.

**Figure 1:**
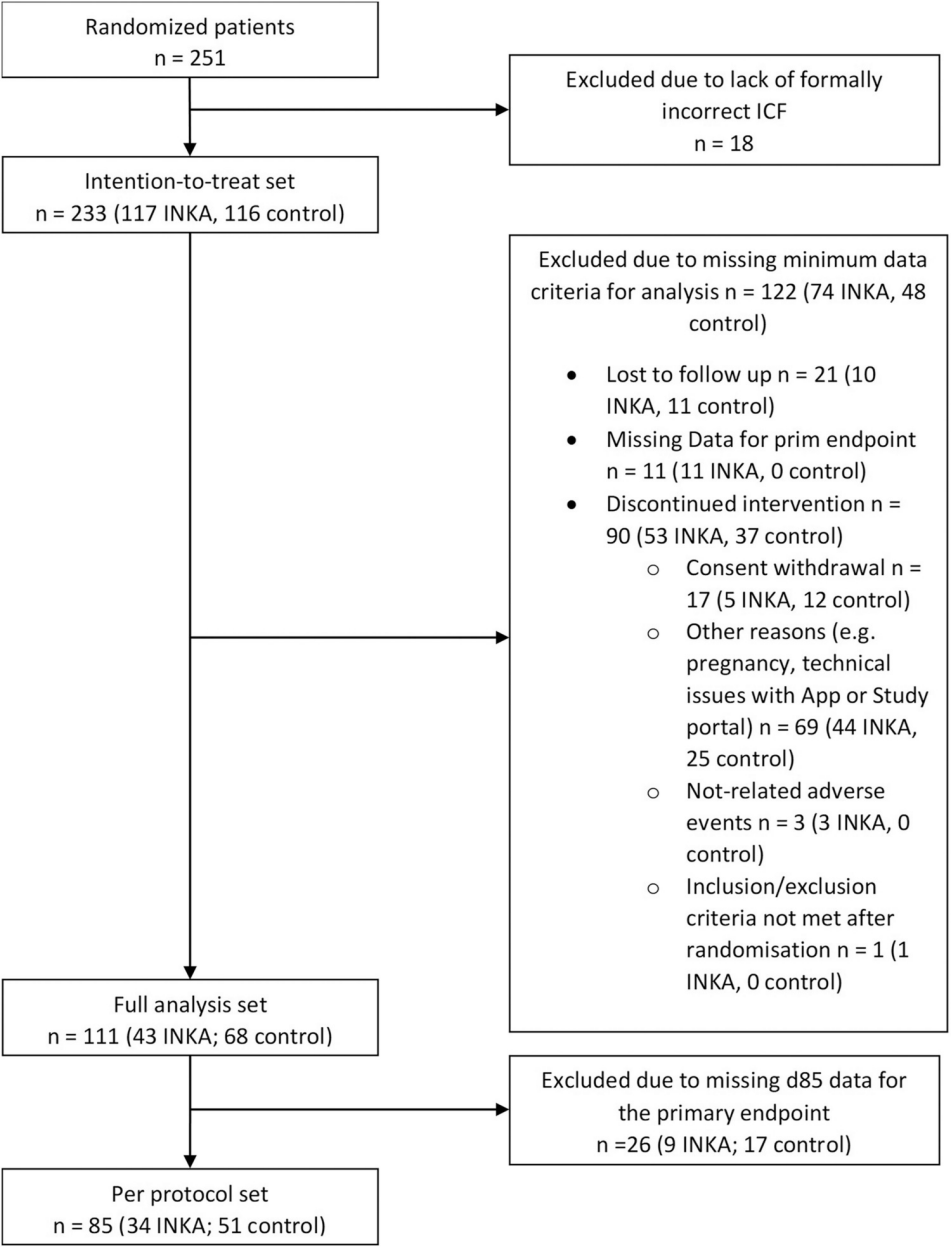
Patient flow chart. ICF, informed consent form.

Out of the 251 patients randomized into the study, 18 were excluded due to a lack of formally correct informed consent (Figure 1). Of the 233 patients in the intention-to-treat (ITT) population, 117 patients were allocated to the INKA group, and 116 patients to the control group. Of these, 10 INKA group patients and 11 control group patients were lost to follow-up. 11 INKA group patients were excluded from the FAS due to missing data for the primary endpoint at baseline, week 4, and/or week 12. Furthermore, 53 INKA group patients and 37 control group patients discontinued the study before completion due to various reasons. Technical problems with the digital systems contributed significantly to early discontinuations. Login and registration difficulties were identified, primarily related to the web portal rather than the INKA app itself. Besides, participants were discouraged by the lack of confirmation of data entry. In the INKA group, participants faced additional challenges, as they had to use the study-specific web portal and the INKA app concurrently.

Therefore, for a total of 111 patients (43 INKA, 67 control) effectiveness endpoint analyses were conducted. 85 patients completed the study with week 12 assessments, thus constituting the per protocol (PP) set (34 INKA, 51 control). The “FAS OAB wet” cohort comprised 93 patients (33 INKA, 60 control), while the “FAS OAB dry” cohort included 18 patients (10 INKA, 8 control).

### Patient characteristics

Demographic data and baseline characteristics were comparable between the two groups (Table 1). The mean age of the FAS population was 52.7 (SD 14.6) years. 27% of patients were male, while 73% were female. As 44% of patients had other pre-existing and concurrent medical conditions, treatment for these conditions, as well as for the underlying OAB, was continued as required.

**Table 1:** Demographic and baseline characteristics of patients in FAS.

Variable	INKA group (*n* = 43)	Control group (*n* = 68)	Total (*n* = 111)
Gender, male/female (%)	25.6/74.4	27.9/72.1	27.0/73.0
Mean age (years)	52.9 (SD 13.1)	52.9 (SD 15.5)	52.7 (SD 14.6)
Previous and concomitant diseases (%)	44.2	44.1	44.1
Number of micturitions per day (3-day average)	9.67 (SD 3.69)	8.93 (SD 3.18)	9.22 (SD 3.38)
Urine volume per micturition (mL)	194.29 (SD 65.22)	207.69 (SD 68.9)	202.5 (SD 67.47)
ICIQ-OAB total score	7.7 (SD 2.63)	6.78 (SD 2.43)	7.06 (SD 2.49)
ICIQ-OABqol total score	50.48 (SD 23.07)	52.18 (SD 28.18)	51.67 (SD 26.65)
ICIQ-UI-SF total score	7.83 (SD 5.36)	8.07 (SD 4.8)	7.98 (SD 5.01)
Amount of urine loss (PAD Test, g)	13.93 (SD 60.24)	10.68 (SD36.53)	11.99 (SD 46.1)

FAS, full analysis set; SD, standard deviation.

### App usage data

55% of the INKA app users engaged with the app daily, with an average of 2.8 sessions per day and a mean usage time of 12 min per session. Analysis of the integrated monitoring system of the app revealed moderate adherence to symptom-specific behaviour recommendations among users. For instance, only one-third of patients adhered to liquid consumption recommendations, and 30% of users, including 41% of males and 27% of females, did not participate in pelvic floor training at all. Additionally, half of the users did not engage in bladder training, and 41% of patients trained for less than a quarter of the recommended training time. Medication adherence was observed in 65% of male and 81% of female users.

### Primary endpoint: number of micturitions per day

**Figure 2:**
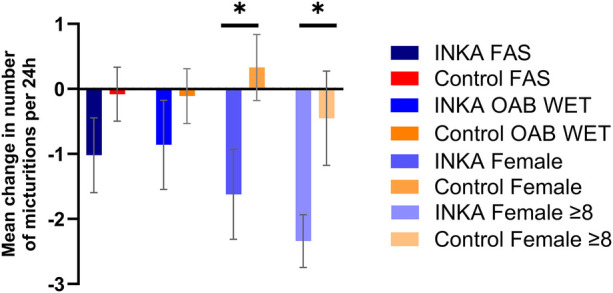
Mean change from baseline in the number of micturitions per 24 h at 12 weeks, presented for Full Analysis Set (FAS; *n* = 85), cohort “OAB wet” (*n* = 73), subgroup females (*n* = 63), and females with 8 micturitions per 24 h at baseline (*n* = 39). Error bars representing standard error of the mean (SEM), * *p* ≤ 0.05.

The primary outcome of the study was the mean change from baseline at 12 weeks in the number of micturitions per 24 h as recorded in the 3-day bladder protocol. After 12 weeks the INKA group reported substantial improvements, experiencing a mean daily decrease in micturitions of −1.02 (SD 3.36; *n* = 34) compared to −0.08 (SD 2.97; *n* = 51) in the control group (*p* = 0.1752) (Figure 2). With LOCF analysis the effect was even more pronounced with a mean reduction of −1.27 (SD 3.08; *n* = 43) in the INKA group vs. −0.21 (SD 2.69; *n* = 68) in the control group, respectively (*p* = 0.0565).

Subgroup analysis of the “OAB wet” and “OAB dry” cohort showed similar tendencies for both groups [OAB wet: - 0.86 (SD 3.05; *n* = 26) INKA group vs. −0.11 (SD 2.88; *n* = 47) control group; *p* = 0.3264; OAB dry: −1.56 (SD 3.0; *n* = 8), + 0.27 (SD 4.38; *n* = 4); *p* = 0.4091]. The subgroup analysis of patients with a higher symptom severity at baseline showed a more pronounced mean symptom improvement for the app user group [−1.92 (SD 3.57; *n* = 21) vs. −0.77 (SD 3.08; *n* = 32) in the control group; *p* = 0.218].

The analysis of the subgroup of females as well as the subgroup of females with a high symptom severity at baseline, revealed statistically significant differences in the mean change of micturitions per 24 h after 12 weeks [females: −1.62 (SD 3.39; *n* = 24) in the INKA group vs. +0.33 (SD 3.18; *n* = 39) in the control group (*p* = 0.0248); females with at least eight micturitions at baseline: −2.78 (SD 3.2; *n* = 16) in the INKA group vs. −0.45 (SD 3.47; *n* = 23) in the control group (*p* = 0.0402)] (Figure 2). Consequently, MCID in this endpoint, reported as a reduction of ≥15%, was reached in both female subgroups (Supplementary Table S1) (34). The subgroup analysis of males showed no statistically significant changes between the treatment groups (data not shown).

### Urine volume per micturition

**Figure 3:**
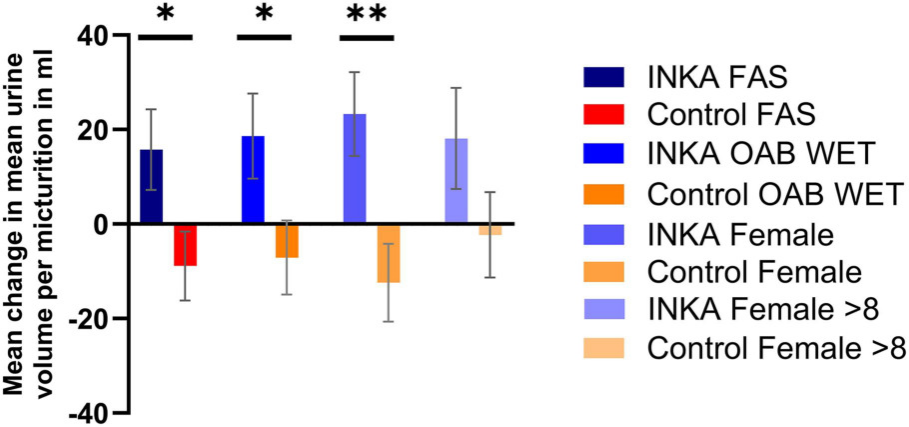
Change from baseline in the mean volume per micturition at 12 weeks, presented for Full Analysis Set (FAS; *n* = 85), cohort “OAB wet” (*n* = 73), subgroup females (*n* = 63), and females with at least 8 micturitions at baseline (*n* = 39). Error bars represent standard error of the mean (SEM), * *p* ≤ 0.05, ** *p* ≤ 0.01.

In correlation with the mean reduction in daily micturitions, a significant increase in the mean urine volume per micturition from baseline was observed at the end of the observation period in the INKA group (+15.75 (SD 49.74) mL; *n* = 34), while the volume decreased in the control group (−8.84 (SD 52.14) mL; *n* = 51) (*p* = 0.0329) (Figure 3). With LOCF analysis the effect was more pronounced with a mean change of +15.83 (SD 48.37; *n* = 43) mL in the INKA group vs. −8.22 (SD 51.12; *n* = 68) mL in the control group, respectively (*p* = 0.0153).

A similar result was obtained for the “OAB wet” cohort with an increase of mean urine volume of +18.63 mL (SD 45.89; *n* = 23) in the INKA group, and decrease of −7.09 mL (SD 53.71; *n* = 47) in the control group (*p* = 0.0431). Results for the “OAB dry” cohort were not significant, most probably due to the limited number of patients in this cohort [+6.39 mL (SD 63.34; *n* = 8) in the INKA group vs. −29.4 mL (SD 21.46; *n* = 4) in the control group (*p* = 0.3069)]. INKA patients who suffered from eight or more micturitions per day at baseline, showed a profound benefit from app usage with an increase of +19.85 mL (SD 45.04; *n* = 21) in the INKA group vs. +0.27 mL (SD 48.17; *n* = 32) in the control group (*p* = 0.1439).

In accordance with the results for the primary endpoint, the female subgroup showed highly significant results as the mean micturition volume increased by +23.29 mL (SD 43.38; *n* = 24) in the INKA group, while it decreased by −12.38 mL (SD 51.5; *n* = 39) in the control group (*p* = 0.0063) (Figure 3). Female patients with 8 or more micturitions at baseline likewise benefited from the digital intervention, but not to the same extent (+18.13 mL (SD 42.68; *n* = 16) in the INKA group vs. −2.28 mL (SD 43.39; *n* = 23) in the control group (*p* = 0.1541) (Figure 3).

Further data recorded in the bladder protocol, including the intensity of urge, the number of diurnal and nocturnal micturitions, and the number of urine leakages per day, did not demonstrate statistical superiority of the INKA group over the control group.

### ICIQ-OAB

The ICIQ-OAB questionnaire, which measures the four OAB-related parameters urinary frequency, urge to urinate, urge incontinence and nocturia symptoms, was used to assess symptom burden and treatment satisfaction from the patients' perspective (25, 26).

**Figure 4:**
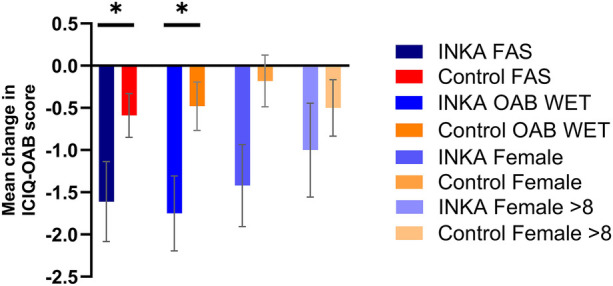
Mean change from baseline in the ICIQ-OAB total score at 12 weeks, presented for Full Analysis Set (FAS; *n* = 82), cohort “OAB wet” (*n* = 72), subgroup females (*n* = 59), and females with at least 8 micturitions at baseline (*n* = 40). Error bars represent standard error of the mean (SEM), * *p* ≤ 0.05.

Following the 12-week treatment period, a significant reduction in the total symptom score of the ICIQ-OAB was reported by the INKA group compared to the control group, with scores of −1.61 (SD 2.27; *n* = 23) and −0.59 (SD 1.98; *n* = 59), respectively (*p* = 0.049) (Figure 4). Similar results were obtained for the INKA “OAB wet” group [−1.75 (SD 2.38; *n* = 20) INKA vs. +-0.48 (SD 2.06; *n* = 52) for the control group; *p* = 0.0283]. The “OAB dry” cohort did not yield significant results which was most likely due to the limited number of patients in this cohort. Analysis of the subgroup of patients with heightened symptom severity at baseline showed a trend towards higher improvement in the INKA compared to the control group.

Female INKA patients reported statistically relevant outcome benefits, with a score reduction of −1.42 (SD 2.12; *n* = 19) compared to −0.18 (SD 1.93; *n* = 40) in the control group (*p* = 0.0287). The female subgroup with heightened symptom severity at baseline showed a clear trend towards better outcomes in the ICIQ-OAB score (−1.0 (SD 2.08; *n* = 14) vs. −0.5 (SD 1.7; *n* = 26; *p* = 0.4172). The clinical importance of the therapeutic benefit of the digital therapy shown for the overall app user group and female subgroup in this score is underlined by a reported MCID of −1.0 for this questionnaire (35).

### ICIQ-OABqol

To investigate whether regular INKA usage may have a positive impact on daily living, enrolled patients completed the condition-specific quality of life questionnaire ICIQ-OABqol (27–29). A positive trend towards more pronounced improvements from baseline to the end of the observation period were observed in the overall INKA group and female subgroup compared to the control groups, although these differences did not reach statistical significance [−12.5 (SD 20.17; *n* = 22) in the INKA group vs. −7.89 (SD 20.15; *n* = 62) in the control group (*p* = 0.359) and −13.56 (SD 19.22; *n* = 18) in the female INKA group vs. −8.72 (SD 22.29; *n* = 43) in the female control group (*p* = 0.4254)].

### Pad test

To quantify the impact of INKA on the severity of incontinence, the standardized one-hour pad test was used (8, 31, 36). INKA users showed a mean decrease of urine loss from baseline to week 12 of −20.05 grams (SD 80.89; *n* = 22) compared to the control group with +0.78 grams (SD 38.26; *n* = 51; *p* = 0.2596). The effect was even more pronounced in the “OAB wet” cohort with a change of −24.5 grams (SD 89.26; *n* = 18) in the INKA group vs. + 0.88 grams (SD 41.77; *n* = 43) in the control group (*p* = 0.2617), in the subgroup of female patients −24.94 grams (SD 94.76; *n* = 16) in the INKA group vs. +0.75 grams (SD 45.72; *n* = 36) in the control group (*p* = 0.3166), and in patients with heightened symptom severity −26.75 grams (SD 94.72; *n* = 16) in the INKA group vs. −0.03 grams (SD 39.9; *n* = 34) in the control group (*p* = 0.293). The greatest effect was observed in female patients with a high symptom severity at baseline, where urine loss decreased by −32.08 grams (SD 109.58; *n* = 13) in the INKA group compared to −0.04 grams (SD 48.86; *n* = 23) in the control group (*p* = 0.3522).

### ICIQ-UI-SF

The ICIQ-UI SF questionnaire, a patient reported outcome tool to assess the impact of symptoms on quality of life, revealed no relevant differences between the changes in the two treatment groups (data not shown) (30). Scores at baseline indicated a relatively low symptom severity in the study patients, with a mean score of 7.83 (SD 5.36; *n* = 42) in the INKA group and 8.07 (SD 4.8; *n* = 68) in the control group, both considered “moderate” according to the recognized rating scale.

### Safety analysis

None of the 13 reported adverse events (AEs) were considered related to the intervention with the INKA app. No device deficiencies were observed.

## Discussion

This first proof-of-concept, randomized, controlled clinical study aimed to investigate the potential of the digital therapy companion INKA in relieving the symptoms of therapy refractory OAB patients. Consistent with current European guideline recommendations (EMEA CPMP/EWP/18/01/Rev. 1), the study examined the reduction in micturition frequency and changes in micturition volume, both recognized as key factors in the symptom burden of OAB patients (37). The decrease in micturitions in the INKA group, accompanied by a significant increase in urine volume per micturition, clearly demonstrated the therapeutic benefit of the digital therapy over a 12-week treatment period.

In this study, INKA achieved an additional benefit in terms of daily micturition frequency, with a reduction of −1.0 in the overall study population and up to −2.7 micturitions in the female subgroup with heightened symptom burden. A lifestyle intervention combined with pelvic floor muscle training (PFMT) in a study by Bykoviene et al. resulted in a reduction of daily micturitions by −1.3 in therapy-naive patients, while another study by Burgio et al. showed a reduction of −2.9 (30, 38). The efficacy of anticholinergic therapy was confirmed in a meta-analysis with an average reduction of −2.06 micturitions per day, associated with the risk of therapy discontinuation due to side effects (39). Compared to these interventions, the use of INKA showed a comparable efficacy in this study, but in patients who were already receiving stable pharmacotherapy and therefore must be considered as refractory patients due to persisting symptoms. Thus, INKA meets patients' need for an additional symptomatic improvement.

Since OAB is recognized as a chronic condition, current research emphasizes the importance of the patient perspective on symptom control (40). Frankel et al. identified a threshold of ≥15% reduction in daily micturitions, correlating with a perceptible improvement in symptoms from patients' perspective (34). Notably, the application of INKA achieved the MCID in the female group and in their subgroup with heightened symptom burden. These results were confirmed by the ICIQ-OAB questionnaire with a mean reduction of −1.61 scores for the whole study population, which extends the recognized clinically relevant improvements, as reported by Verghese et al. (35). Further, MCID were achieved in the “OAB wet” and female subgroup.

In addition, INKA was able to achieve additional enhancements in health-related quality of life (HRQoL) which is known to be substantially elevated by pharmacological treatment of OAB (41). Urinary incontinence improved in all INKA and subgroup patients, particularly in females with heightened symptom burden, although compliance to PFMT could be further improved. This will be addressed by implementing reminders for patients through push notifications into the upcoming version INKA 2.0. However, the improvement of urinary incontinence was not reflected in the ICIQ-UI-SF questionnaire score, possibly due to the moderate patient perceived burden at baseline.

Overall, the “OAB wet” patient subgroup, patients with higher symptom burden, and especially females benefited most in the examined endpoints from using INKA. The dashboard analysis showed that females adhered the most to medication intake and pelvic floor training specifications, whereas males showed lower adherence, which might have contributed to the extended benefit observed. In general, the data for medication intake demonstrated an increased adherence of INKA users compared with reported adherence rates (42).

We conclude that the application of INKA provides additional benefits for patients under first-line pharmacotherapy, while its use in therapy-naïve patients could yield substantial advantages, potentially increasing further if patients show high adherence to the digital therapy program guidelines. Given the proven safety of INKA and the positive outcomes of this study, the benefit-risk ratio for this intervention is favourable.

Other digital self-management solutions for OAB treatment have also proven positive results. In the USA, the digital therapy CeCe effectively reduced symptoms in therapy-naïve patients in first-line therapy (35). An app-based intervention developed in Sweden improved urgency and incontinence in females (43). To our knowledge, however, INKA is the first digital solution for mobile devices in the German-speaking region to provide effective, comprehensive behavioral therapy for both female and male OAB patients, including those with ongoing first-line pharmacotherapy needs.

The healthcare benefits of INKA also include promoting everyday health activities of OAB patients and facilitating processes between patients and care providers. OAB places an estimated annual burden of 3.98 billion Euros on the German health and social system, with the majority of costs arising from medical visits and treatments (44, 45). Given the demonstrated medical benefits of INKA in this study, which are likely to be transferable to routine clinical practice, combined with the minimal time investment for patients and the positive socioeconomic balance, integration of INKA into routine care seems warranted.

Several limitations were identified during the study. Due to the nature of the intervention, study participants were not blinded, which may have positively influenced the assessment of the intervention group. A relatively high dropout rate was observed, mostly due to technical difficulties, that were encountered by INKA participants more frequently than by control participants Login and registration issues were naturally more pronounced in the INKA group due to the use of two platforms. Further, data integrity may have been influenced by missing or merged data entries, which arose from technical failures in the beginning of the study. These represent common challenges encountered in digital health studies (46). Although, the therapeutic INKA program was not affected, these difficulties may have affected internal validity. Patients only had moderate symptom burden at baseline, making it challenging to achieve statistical significance in certain endpoints. The results for diurnal and nocturnal micturitions were not statistically significant, which will be investigated in the next study. Lastly, 30–50% of enrolled patients did not entirely follow the symptom-specific recommendations, which will be addressed in the follow-up study with more detailed instructions and integration of push messages in the INKA 2.0 version.

## Conclusion

The digital therapy companion INKA is an innovative home application that offers clinical benefits to OAB patients even while undergoing first-line pharmacotherapy. Future studies aim to validate these results using an improved version, INKA 2.0, in a randomized controlled study with a more streamlined patient population exhibiting higher symptom burden at study start.

Trademark: INKA; registration number: 018614110 (Dr Pfleger Arzneimittel GmbH, Bamberg, Germany).

## Data Availability

The datasets used and/or analyzed during the current study as well as the trial protocol and/or statistical analysis plan are available from the sponsor of the study, NEXTEC medical GmbH, upon reasonable request. (info@nextec-medical.com).
