# Supplementary material for: A randomized, unblinded, controlled clinical study to assess the mobile digital health application INKA in the management of therapy refractory overactive bladder and mixed incontinence
Source: Front Digit Health. 2026 Apr 10;8:1610663. doi: 10.3389/fdgth.2026.1610663 (PMC13106321; doi:10.3389/fdgth.2026.1610663)

**A Randomized, Open-label, Controlled Clinical Study to Assess the Mobil Digital Health Application INKA in the Management of Therapy Refractory Overactive Bladder and Mixed Incontinence**

Submitted to: Frontiers in Digital Health

| Item No. | Reply | Manuscript Page |
| --- | --- | --- |
| 1a | i) “Mobil” as the mode of delivery is indicated in the title.  ii) There are no co-interventions.  iii) The primary condition or target group is “Therapy Refractory Overactive Bladder and Mixed Incontinence” which is mentioned in the title. | Title |
| 1b | i) The key features/functionalities/components of the intervention and comparator are mentioned in the abstract as follows: “INKA offers self-guided educational, behavioural, and motivational content in accordance with current clinical guidelines, along with physiotherapy modules including guided bladder training and pelvic floor exercises. Tracking tools for fluid intake, micturition frequency, and reminders on medication intake, further support daily OAB management in the home setting.”.  “251 patients under first-line stable pharmacological treatment were recruited at 35 study sites in Germany and randomized to receive access to the INKA app or to receive standard of care (control group).”  ii) The INKA app is a self-management tool for patients, without involvement of medical professionals. This is indicated in the abstract as follows: “Tracking tools for fluid intake, micturition frequency, and reminders on medication intake, to support daily patient self-management of OAB in the home setting.”  “Self-assessed OAB related endpoints were investigated at baseline, after 4 and after 12 weeks.”  iii) Participants were recruited in 35 study sites across Germany. This is mentioned in the abstract as follows: “251 patients under first-line stable pharmacological treatment were recruited at 35 study sites in Germany”.  Screening and end-of-study visit were performed at the study sites. Baseline, week 4, and week 12 assessments were self-assessed through questionnaires. This is mentioned in the abstract as follows:  “251 patients under first-line stable pharmacological treatment were recruited at 35 study sites in Germany and randomized to receive access to the INKA app or to receive standard of care (control group). Self-assessed OAB related endpoints were investigated at baseline, after 4 and after 12 weeks. The end-of-study visit was conducted at the study sites.”  We used “unblinded” to indicate the level of blinding in the title and the abstract (“This exploratory, two-arm, randomized, unblinded, controlled, multicentre study assessed the health benefits of INKA…”)  iv) The abstract contains the number of enrolled and analysed participants in each group, as well as the percentage of daily use of the intervention:  “251 patients under first-line stable pharmacological treatment were recruited…”, “For the 111 evaluable patients (43 INKA, 68 control), baseline characteristics were comparable…”, “55.4% of INKA users engaged with the app daily.”  v) The primary outcome is discussed in the Conclusion section of the abstract:  “This proof-of-concept study highlights the potential of the INKA mobile app to reduce the micturition frequency and increase the micturition volume in therapy refractory OAB patients, both recognized as key factors of OAB symptom burden.” | Title, Abstract |
| 2a | i) Given the limited therapeutic options for many OAB patients, INKA was developed as a cost-effective therapeutic option with a good efficacy profile and minimal side effects. INKA can be utilized as a standalone intervention in treatment-naive patients to enhance the impact of conservative strategies or it can be used as an add-on therapy to pharmacological treatments.  ii) This is the first proof-of-concept study to investigate the medical benefit of INKA in OAB patients.  iii) The comparator is standard-of-care alone. | Introduction, Intervention |
| 3b | At study initiation, intermittent system failures and downtimes occurred in both digital components (study web portal and INKA app), necessitating several bug fixes. Urgent technical issues were addressed via hot fixes within ≤3 days after identification. These fixes were limited to system availability, and the reliable delivery of automated emails and notifications. No changes were made to the study methodology, therapeutic content, intervention logic, or comparator during the trial period. Apart from the early technical issues described, no further unexpected events (e.g., staff changes or prolonged system outages) occurred that influenced study conduct. |  |
| 4a | i) The inclusion criteria required that participants possess a smartphone and be proficient in its use, including the ability to respond to push notifications.  ii) see 1b. This information is implemented in the Methods section as well.  iii) Patients were briefed at the study site verbally and received the written informed consent form. The information contained all trial related activities including trial objectives, voluntary participation and the right to withdraw at any time without consequences, in- and exclusion criteria, randomization procedures, timely commitment and study duration, potential benefits and risks, reporting of concomitant medication, remuneration, insurance coverage, data collection, data storage and assessment, as well as data protection measures.  Unique to this trial, participants were explained the two treatment groups. Patients were informed that the use of INKA is an add-on to standard of care, while the control group remains with standard-of-care. Further, patients were explained how to access the INKA app and web portal. | Patients, Recruitment and Randomization, Collection and Handling of Data, Sample Size and statistical methods |
| 4b | i) All outcomes were self-assessed. This is indicated in the Methods/Study outcome section.  ii) Not applicable, as recruitment was performed in person at 35 study sites in Germany. | Collection and Handling of Data, Sample Size and statistical methods |
| 5 | i) DataArt GmbH, Landshuter Allee 10, 80637 Munich, Germany, is the developer of INKA. This is indicated in the Methods section.  Further information on sponsoring, ownership, and commercial distribution is provided in the “Competing interests” section:  “NEXTEC medical GmbH, Zöllinplatz 4, 79410 Badenweiler, Germany, is the manufacturer and owner of the INKA app, as well as the sponsor of the study. Dr. Pfleger Arzneimittel GmbH, Dr.-R.-Pfleger-Str. 12, 96052 Bamberg, Germany, is the commercial distributor of the INKA app and funded the study.”  ii) Development complied with IEC 62304 for software development and ISO 13485 Design Control. Usability was tested in accordance with IEC 62366 (formative and summative evaluation).  Two usability tests were conducted with users of different ages and genders on the majority of features. These included the registration process, the onboarding process, the user profile, the notification settings, the medical questions, the home screen, the chart view, the navigation menu, the daily trackers, the medication trackers, the urge menu, the gamification, the urine pad test, the micturition protocol, the education, and the report.  iii) The version number of the INKA app used in this study is “1.0”. This is indicated in the Methods section/Intervention. The content of the INKA app was “frozen” during the trial. The bug fixes performed were not related to the therapeutic content, as described in 3b. INKA does not contain news feeds or changing content.  iv) At trial start, INKA was CE-marked under the EU MDR. Accuracy and quality of all user-facing information were governed by the ISO 13485–compliant QMS of the manufacturer, including documented content governance with clinical sign-off, version control and change management, risk management (ISO 14971), usability engineering (IEC 62366), and software life-cycle verification/validation (IEC 62304). Releases followed predefined test plans with requirements-to-test traceability and formal release approval. MDR post-market surveillance and vigilance processes were active during the study. Only the approved production version was deployed to participants.  v) Due to its status as a commercial, CE-marked medical device, the INKA source code and proprietary algorithms cannot be shared, and parts of the content are subject to ongoing IP protection. To support replicability, a detailed description of the intervention components (Methods section) and selected screenshots are provided.  vi) The current production version of the INKA application (used in a subsequent study) is available at https://www.meine-inka.de. The version evaluated in the present trial is no longer available in the app stores. Therefore, screenshots are provided as supplementary file 1.  vii) The participants received a QR code at the study site. This QR code enabled both intervention and control participants to access the web portal. In addition, participants in the intervention group were able to download the INKA app via the QR code. The INKA app was provided free of charge. Participants were screened by the investigators according to the in- and exclusion criteria stated in the study protocol. Selection of participants was performed according to the in- and exclusion criteria. Access to the INKA app for editors and reviewers can no longer be provided as only the latest version of the application is available.  viii) INKA is a smartphone-based digital therapeutic delivered via a mobile application. Its features, functionalities, and components are described in detail in the Methods (Intervention), including modular educational content, pelvic floor training and bladder-training exercises, reminders, and progress tracking. Content is evidence based and aligned with current clinical guidelines; sources and responsible clinical experts are referenced in the manuscript. The intervention is tailored to individual symptom status, as users report baseline and ongoing symptoms that adopt bladder training intensity/length, and reminder schedules. Users can track their progress over time via in-app summaries and receive automated feedback on adherence and symptom trends. Behavioral change techniques include education and self-monitoring.  INKA does not include a direct physician–patient communication channel. However, patient-reported data can be exported as a PDF for discussion during routine medical visits (not part of the study).  Presentation follows usability and accessibility principles appropriate for a CE-marked medical device (MDR class I), with concise text blocks, visual cues, and structured navigation; no external hyperlinks are required for core functions. Current functionality remains publicly described at [https://www.meine-inka.de](https://www.meine-inka.de/).  ix) INKA usage was individualized and automatically adapted to current symptom severity as assessed in-app (ICIQ-UI SF) and ranged between 25 and 50 minutes. Based on this assessment, the app recommended the timing, frequency, and intensity of modules (education, pelvic floor exercises, bladder training), rather than ad libitum use. Patients received push notifications for medication intake, email reminders for bladder diaries and questionnaires, and in-app notifications providing behavioral feedback (e.g., fluid intake >1.5 L/day, >2 coffees/day), daily medication completion, and weekly adherence summaries. Clear instructions and demonstrations for correct pelvic floor exercise execution and bladder training were provided within the app. All use parameters and instructions are detailed in the Methods/Intervention section.  x) The INKA app is a self-management tool for patients to be used in the home setting. In routine medical practice, INKA is recommended to patients by their treating physicians. For doctor´s appointments the data recorded in the app can be exported as a pdf file to make it available to the treating physician.  In this clinical trial, trial participants were recruited at medical practices. Further, the end-of-study visit was performed at the study sites.  xi) Prompts are solely automatically performed by the INKA app, comprising the following situations: Reminders to take medication, to document fluid intake, to perform bladder training and pelvic floor exercises. Via the urge button, INKA prompts bladder training sessions to delay voiding in acute situations. Further, an in-app reward system motivates patients to engage daily with INKA.  INKA is used in routine settings in the same manner as in this clinical trial.  xii) INKA is a standalone intervention. No co-interventions were used in this clinical trial. No co-interventions are used in routine medical care. | Trial design and trial data, Competing interests |
| 6a | i) The online questionnaires used are all validated and frequently used in clinical trials. As it is well recognized that electronic versions would require re-validation, the ICIQ has not yet performed these validations. Therefore, in this study the ICIQ recommendation was followed. The questionnaires were supplied in the electronic format as similar to the original paper document as possible (https:// iciq.net).  The usability and technical functionality of the electronic questionnaires had been tested prior to study initiation.  ii) Use was objectively defined and monitored via background app usage logs, capturing frequency of use, accessed functionalities, number of sessions, and time engaged per session. These data were analysed descriptively as process outcomes, including daily engagement rates, average sessions per day, and engagement time. Usage metrics were collected separately from the clinical trial database and did not inform clinical outcome analyses. The data will not be provided as it is not part of the study.  Intervention content and training modules were developed in collaboration with a rehabilitation medicine specialist in pelvic floor therapy and reviewed with leading urologists; they were assessed within the MDR clinical evaluation and raised no safety or quality concerns.  iii) No qualitative data was collected. | Collection and Handling of Data, Results |
| 7a, b | Due to the exploratory nature of this proof-of-concept study formal hypothesis testing was not performed. An initial sample size of 216 patients was estimated to be sufficient to address the primary objective. After performance of the exploratory interim analysis the sample size was adjusted to 250 patients. |  |
| 8a, 8b | The random allocation sequence was generated using a computer-based random number algorithm (SAS® 9.4). The randomization was automatically performed via the study’s eCRF. The randomization list is an electronic file.  Randomization Ratio: 1:1 with UI, 1:1 without UI  Total number of Blocks: 100, block size: 4  Stratification: yes  Variable: gender  Levels: 1 male, 2 female  The electronic randomization list was stored in the eCRF and was not visible to the investigators/study site staff. |  |
| 9 | see above (8a, 8b) |  |
| 10 | The randomization list was prepared by the responsible statistician of the Contract Research Organization.  The participants were enrolled at the study sites (urological practices, clinics).  The assignment to interventions was performed automatically by the eCRF after patient data was entered at the study site. |  |
| 11a | i) Due to the nature of the intervention all study participants were unblinded. Site personnel were unblinded either.  No co-interventions were used.  ii) During the informed consent process, patients were informed that the intervention was a digital app to support OAB treatment and that the control group would receive standard of care. | Recruitment and Randomization |
| 11b | n.a. |  |
| 12a | i) Data was analysed descriptively. No adjustment for multiple testing took place. Missing data were not replaced. Attrition rates are shown in Figure 1 (Patient Flow Chart), as well as the number of patients included in FAS. FAS consisted of all patients with completed baseline assessments and with at least one post baseline assessment regarding the primary outcome. LOCF was performed for sensitivity analyses only. | Sample Size and statistical methods, Results |
| 12b | Subgroup analyses were performed in the same manner as for the total cohort on all outcome parameters. | Sample Size and statistical methods |
| X26 | i) This is included in section Methods/Trial design and trial data  ii) Informed consent was obtained offline at the study sites. See 4a ii) for the summary of information provided to study participants.  iii) The patient information included details of the potential benefits and risks, as well as detailed information on data protection. | Recruitment and Randomization, Trial design and trial data |
| 13a | The distribution of participants (randomized, treated, analysed for the primary outcome) are displayed in Figure 1. As previously explained the patients were automatically randomly assigned to the control and intervention group via the eCRF. Depending on the time of study inclusion, patients were assigned to groups independently of the study site.  The study was conducted at 35 study sites in Germany. | Results |
| 13b | Figure 1 displays the number of participants who were excluded from the analysis with the corresponding reasons. | Results |
| 14a | No critical “secular event” happened during conduct of the trial. |  |
| 14b | The exploratory interim analysis led to an adjustment of the sample size to 250 participants. The trial was discontinued after the target number was reached. | Sample Size and statistical methods |
| 15 | i) A table with baseline characteristics of FAS participants is included in the Results/Recruitment and baseline characteristics section. Participants were selected based on proficiency in smartphone use during screening. | Table 1 |
| 16 | i) Figure 1 displays the number of patients who consented in trial participation (n = 251) and the number of participants in the intention-to-treat set (n = 233). Effect sizes were not determined. Data for the primary and secondary endpoints were summarized by group and time point.  App usage data is provided in the Results/App usage data section.  ii) The aim of this proof-of-concept study was to gain preliminary insights into patient acceptance of the digital intervention and the health benefit of using INKA in the patient population studied. A series of technical issues (login and registration difficulties) that were encountered after study initiation, led to an increased dropout rate. Therefore, FAS was chosen rather than ITT for primary and secondary analyses. | Figure 1 |
| 17a | i) App usage data is provided in section Results/App usage data and in section Methods/Intervention. Session was defined as the duration with continuous interaction with the app, i.e. the time when INKA was opened until INKA was closed or moved to the background. | Collection and Handling of Data, App usage data |
| 17b | n.a. |  |
| 18 | n.a. | Collection and Handling of Data |
| 19 | i) Technical problems with usage of the web portal and the INKA app were encountered mostly during early recruitment and were mostly related to login and registration issues. These technical issues were fixed during the study. No privacy breaches were encountered.  ii) No qualitative data was collected. | Collection and Handling of Data, Recruitment and baseline characteristics |
| 22 | i), ii) Study findings, unanswered questions and suggestions for future research are examined in the section Discussion. | Discussion |
| 20 | Potential biases related to unblinding, the increased drop-out rate, missing or merged data, the non-adherence of participants to the app instructions are addressed in the Discussion section. Expectation bias due to the informed consent procedure can be considered excluded, as at the time of informed consent patients were unaware of the future group allocation. | Discussion |
| 21 | i) INKA is intended to be used by OAB patients with or without urinary incontinence only. Given the demonstrated medical benefit of INKA in this study, we are convinced that the results can be transferred to routine clinical practice.  ii) In this RCT INKA was used in the same manner as it would be used in a routine clinical setting. | Results |
| 23 | This is indicated in the Methods/Trial design and trial data section. | Trial design and trial data |
| 24 | Further information can be found at the following study registry: [DRKS - Deutsches Register Klinischer Studie, https://www.drks.de/search/de/trial/DRKS00029329/details](DRKS%20-%20Deutsches%20Register%20Klinischer%20Studie,%20https:/www.drks.de/search/de/trial/DRKS00029329/detailsn) |  |
| 25 | This is indicated in the section Competing interests. | Competing interests |
| X27 | This is indicated in the section Competing interests. | Competing interests |

**Supplementary file 1: Screenshots INKA app 1.0**


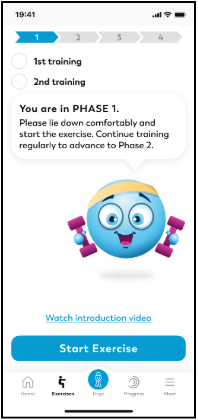

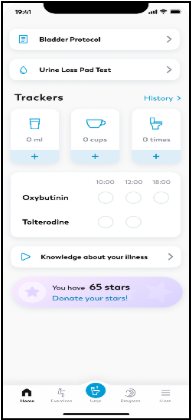

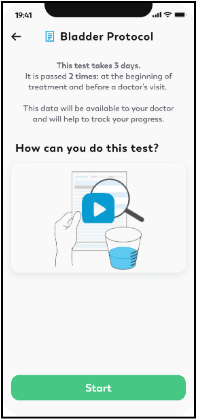

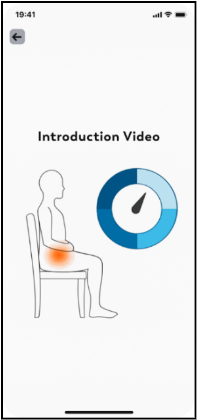

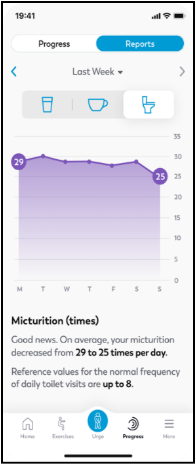

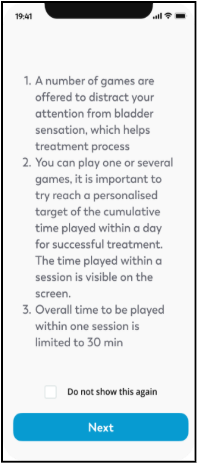

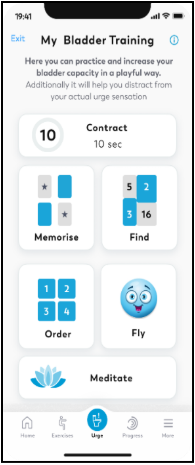

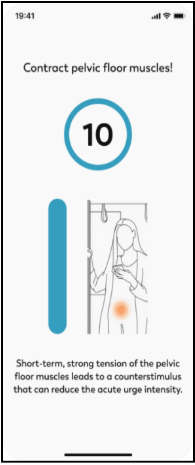

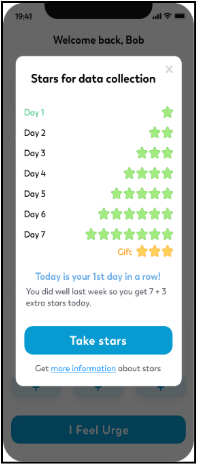

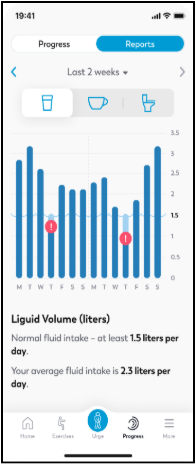

Supplement: Supplementary file 1 [file Datasheet1.docx]
