# Supplementary material for: A randomized, unblinded, controlled clinical study to assess the mobile digital health application INKA in the management of therapy refractory overactive bladder and mixed incontinence
Source: Front Digit Health. 2026 Apr 10;8:1610663. doi: 10.3389/fdgth.2026.1610663 (PMC13106321; doi:10.3389/fdgth.2026.1610663)
Supplement: Supplementary file 2 [file Datasheet2.pdf]

Table S.1: Number of Micturitions per Day (Average from 3-day Bladder Protocol)
Full Analysis Set

	*)	n	Mean (SD)	Median	Q1 , Q3	Min , Max	Change from Baseline (T1) n	Mean (SD)	Median	Q1 , Q3	Min , Max
INKA	T1	43	9.67 (3.69)	8.77	6.8 , 11.7	4.3 , 20.7					
	T2	26	8.76 (4.35)	7.63	6.1 , 8.9	4.7 , 24.6	26	-0.09 (4.65)	-1.01	-3 , 0	-4.5 , 16.1
	T3	34	8.93 (3.64)	8.77	6.8 , 10.6	3.1 , 21.9	34	-1.02 (3.36)	-0.86	-3.5 , 0.5	-11.1 , 6.5
	T2, LOCF	43	9.62 (4.52)	8.15	6.5 , 12.7	4.3 , 24.6	43	-0.05 (3.59)	0	-1.4 , 0	-4.5 , 16.1
	T3, LOCF	43	8.39 (3.43)	7.93	5.9 , 10	3.1 , 21.9	43	-1.27 (3.08)	-1.23	-3.4 , 0.3	-11.1 , 6.5
Control	T1	68	8.93 (3.18)	8.82	7.4 , 10.6	2.2 , 17.7					
	T2	54	8.85 (2.85)	8.75	6.9 , 10.9	2.5 , 17.2	54	-0.36 (2.53)	-0.21	-2.1 , 1.2	-6 , 4.9
	T3	51	8.87 (3.39)	8.12	6.3 , 11.3	2.6 , 18.8	51	-0.08 (2.97)	-0.08	-2.6 , 1.9	-6.4 , 7.4
	T2, LOCF	68	8.64 (2.96)	8.62	6.5 , 10.9	2.5 , 17.2	68	-0.29 (2.25)	0	-1.5 , 0.6	-6 , 4.9
	T3, LOCF	68	8.72 (3.31)	8.01	6.5 , 10.9	2.5 , 18.8	68	-0.21 (2.69)	-0.26	-2.3 , 1.2	-6.4 , 7.4

The difference of the change from baseline after 12 weeks between the 2 groups was tested using the 2-sample t-test: P-value = 0.1752
The difference of the change from baseline after 12 weeks (LOCF) between the 2 groups was tested using the 2-sample t-test: P-value = 0.0565

*) T1 = Baseline ; T2 = Week 4 ; T3 = Week 12

Table S.2: Number of Micturitions per Day (Average from 3-day Bladder Protocol)
Full Analysis Set - Subpopulation Analysis including all **OAB Wet** Patients

							Change from Baseline (T1)				
	*)	n	Mean (SD)	Median	Q1 , Q3	Min , Max	n	Mean (SD)	Median	Q1 , Q3	Min , Max
INKA	T1	33	9.55 (3.78)	9.09	6.8 , 10.4	4.3 , 20.7					
	T2	20	8.18 (3.0)	7.63	6 , 8.6	4.9 , 16.4	20	-0.38 (3.54)	-1.01	-2.7 , -0.1	-3.7 , 9.1
	T3	26	8.82 (4.06)	8.71	5.8 , 10.6	3.1 , 21.9	26	-0.86 (3.5)	-1.15	-3.1 , 0.5	-11.1 , 6.5
	T2, LOCF	33	9.32 (4.16)	8.05	6.5 , 11.3	4.3 , 20.7	33	-0.23 (2.74)	0	-1.3 , 0	-3.7 , 9.1
	T3, LOCF	33	8.36 (3.74)	7.73	5.8 , 9.8	3.1 , 21.9	33	-1.19 (3.2)	-1.24	-3.3 , 0	-11.1 , 6.5
Control	T1	60	8.84 (3.2)	8.68	7.4 , 10.2	2.2 , 17.7					
	T2	47	8.81 (2.66)	8.8	6.9 , 10.9	2.5 , 14.4	47	-0.33 (2.62)	-0.12	-2.1 , 1.2	-6 , 4.9
	T3	47	8.7 (3.19)	8.04	6.3 , 11.3	2.6 , 14.4	47	-0.11 (2.88)	-0.02	-2.7 , 1.9	-6.4 , 7.4
	T2, LOCF	60	8.58 (2.85)	8.62	6.4 , 10.9	2.5 , 14.4	60	-0.26 (2.31)	0	-1.4 , 0.8	-6 , 4.9
	T3, LOCF	60	8.57 (2.99)	8.01	6.5 , 10.9	2.5 , 14.4	60	-0.27 (2.64)	-0.25	-2.3 , 1.2	-6.4 , 7.4

The difference of the change from baseline after 12 weeks between the 2 groups was tested using the 2-sample t-test: P-value = 0.3264
The difference of the change from baseline after 12 weeks (LOCF) between the 2 groups was tested using the 2-sample t-test: P-value = 0.1373

*) T1 = Baseline ; T2 = Week 4 ; T3 = Week 12

Table S.3: Number of Micturitions per Day (Average from 3-day Bladder Protocol)
Full Analysis Set - Subpopulation Analysis including all **OAB Dry** Patients

							Change from Baseline (T1)				
	*)	n	Mean (SD)	Median	Q1 , Q3	Min , Max	n	Mean (SD)	Median	Q1 , Q3	Min , Max
INKA	T1	10	10.07 (3.55)	8.57	7.7 , 12.7	5.8 , 17.6					
	T2	6	10.7 (7.38)	7.83	6.2 , 13	4.7 , 24.6	6	0.88 (7.67)	-1.63	-3.4 , 0.4	-4.5 , 16.1
	T3	8	9.29 (1.78)	9	8 , 10.7	6.9 , 11.9	8	-1.56 (3.0)	-0.3	-4.5 , 0.8	-5.9 , 1.5
	T2, LOCF	10	10.6 (5.71)	8.67	6.9 , 12.8	4.7 , 24.6	10	0.53 (5.73)	0	-3 , 0	-4.5 , 16.1
	T3, LOCF	10	8.53 (2.28)	8.69	6.9 , 10	4.7 , 11.9	10	-1.55 (2.79)	-0.3	-3.5 , 0.4	-5.9 , 1.5
Control	T1	8	9.59 (3.19)	9	7.9 , 12.1	4.4 , 14.5					
	T2	7	9.08 (4.2)	7.23	6.6 , 11.8	4.9 , 17.2	7	-0.57 (1.96)	-0.29	-2.3 , 0.5	-3.2 , 2.8
	T3	4	10.76 (5.51)	9.05	7.4 , 14.2	6.2 , 18.8	4	0.27 (4.38)	-1.54	-2.5 , 3	-2.5 , 6.7
	T2, LOCF	8	9.09 (3.89)	8.08	6.8 , 10.5	4.9 , 17.2	8	-0.5 (1.83)	-0.21	-1.8 , 0.3	-3.2 , 2.8
	T3, LOCF	8	9.84 (5.25)	7.73	6.4 , 13.4	4.9 , 18.8	8	0.25 (3.17)	-0.4	-2.4 , 1.7	-2.5 , 6.7

The difference of the change from baseline after 12 weeks between the 2 groups was tested using the 2-sample t-test: P-value = 0.4091
The difference of the change from baseline after 12 weeks (LOCF) between the 2 groups was tested using the 2-sample t-test: P-value = 0.2207

*) T1 = Baseline ; T2 = Week 4 ; T3 = Week 12

Table S.4: Number of Micturitions per Day (Average from 3-day Bladder Protocol)
Full Analysis Set
- Subgroup Analysis by Gender: **Female** -

	*)	n	Mean (SD)	Median	Q1 , Q3	Min , Max	Change from Baseline (T1) n	Mean (SD)	Median	Q1 , Q3	Min , Max
INKA	T1	32	9.94 (4.0)	9.19	7.1 , 12	4.3 , 20.7					
	T2	21	8.33 (3.21)	7.69	5.9 , 8.9	4.7 , 16.4	21	-0.61 (3.61)	-1.03	-3.3 , -0.1	-4.5 , 9.1
	T3	24	8.83 (3.02)	9.02	6.9 , 10.9	4 , 17.1	24	-1.62 (3.39)	-1.33	-3.6 , 0.2	-11.1 , 6.4
	T2, LOCF	32	9.53 (4.22)	8.52	6 , 12.7	4.3 , 20.7	32	-0.4 (2.92)	-0.07	-1.9 , 0	-4.5 , 9.1
	T3, LOCF	32	8.17 (2.9)	7.71	5.9 , 10	4 , 17.1	32	-1.77 (3.02)	-1.5	-3.5 , 0	-11.1 , 6.4
Control	T1	49	9.01 (3.13)	9.17	7.4 , 11.2	2.2 , 17.7					
	T2	39	9.1 (2.79)	9.31	7.3 , 11.1	2.5 , 14.4	39	-0.16 (2.7)	0.22	-2.4 , 1.5	-5.8 , 4.9
	T3	39	9.28 (3.33)	8.45	6.9 , 11.6	3.5 , 18.8	39	0.33 (3.18)	0.31	-2.5 , 2.6	-6.4 , 7.4
	T2, LOCF	49	8.88 (2.83)	9	6.9 , 10.9	2.5 , 14.4	49	-0.13 (2.4)	0	-1.6 , 1.2	-5.8 , 4.9
	T3, LOCF	49	9.06 (3.17)	8.45	7.2 , 11.3	2.5 , 18.8	49	0.05 (2.96)	-0.02	-2.2 , 1.9	-6.4 , 7.4

The difference of the change from baseline after 12 weeks between the 2 groups was tested using the 2-sample t-test: P-value = 0.0248
The difference of the change from baseline after 12 weeks (LOCF) between the 2 groups was tested using the 2-sample t-test: P-value = 0.0089

*) T1 = Baseline ; T2 = Week 4 ; T3 = Week 12

Table S.5: Number of Micturitions per Day (Average from 3-day Bladder Protocol)
Full Analysis Set
- Subgroup Analysis (**patients with a mean number of micturitions over 24 hours of at least 8 at baseline**) -

	*)	n	Mean (SD)	Median	Q1 , Q3	Min , Max	Change from Baseline (T1) n	Mean (SD)	Median	Q1 , Q3	Min , Max
INKA	T1	28	11.43 (3.39)	10.29	8.9 , 12.7	8.1 , 20.7					
	T2	16	9.09 (4.68)	7.96	6.7 , 8.8	4.7 , 24.6	16	-1.22 (4.8)	-2.74	-3.3 , -0.9	-4.5 , 16.1
	T3	21	10.19 (3.73)	9.38	8.6 , 11.5	5.1 , 21.9	21	-1.92 (3.57)	-1.56	-3.6 , -0.2	-11.1 , 6.5
	T2, LOCF	28	10.73 (4.71)	8.82	7.8 , 12.7	4.7 , 24.6	28	-0.7 (3.63)	-0.13	-3 , 0	-4.5 , 16.1
	T3, LOCF	28	9.3 (3.62)	9.02	6.8 , 10.6	4.7 , 21.9	28	-2.13 (3.13)	-2.37	-3.6 , -0.5	-11.1 , 6.5
Control	T1	44	10.63 (2.38)	9.88	8.8 , 12.1	8.1 , 17.7					
	T2	37	9.75 (2.68)	9.52	7.9 , 11.2	4.4 , 17.2	37	-0.87 (2.67)	-0.73	-2.6 , 0.5	-6 , 4.9
	T3	32	10.02 (3.17)	9.95	7.5 , 12.1	4.9 , 18.8	32	-0.77 (3.08)	-0.81	-3.3 , 1.3	-6.4 , 6.7
	T2, LOCF	44	9.9 (2.57)	9.53	8.2 , 11.5	4.4 , 17.2	44	-0.73 (2.47)	-0.39	-2.3 , 0.3	-6 , 4.9
	T3, LOCF	44	9.85 (3.06)	9.53	7.7 , 11.7	4.9 , 18.8	44	-0.78 (2.77)	-0.81	-2.7 , 0.9	-6.4 , 6.7

The difference of the change from baseline after 12 weeks between the 2 groups was tested using the 2-sample t-test: P-value = 0.218
The difference of the change from baseline after 12 weeks (LOCF) between the 2 groups was tested using the 2-sample t-test: P-value = 0.0598

*) T1 = Baseline ; T2 = Week 4 ; T3 = Week 12

Table S.6: Number of Micturitions per Day (Average from 3-day Bladder Protocol)
Full Analysis Set
- Subgroup Analysis (**Female patients with a mean number of micturitions over 24 hours of at least 8 at baseline**) -

	*)	n	Mean (SD)	Median	Q1 , Q3	Min , Max	Change from Baseline (T1): n	Mean (SD)	Median	Q1 , Q3	Min , Max
INKA	T1	22	11.68 (3.59)	10.79	9.1 , 12.8	8.1 , 20.7					
	T2	13	8.15 (2.43)	7.88	6.6 , 8.7	4.7 , 13	13	-2.34 (1.48)	-3	-3.4 , -1	-4.5 , 0
	T3	16	9.82 (2.87)	9.33	8.3 , 11.5	5.1 , 17.1	16	-2.78 (3.2)	-2.74	-3.9 , -0.7	-11.1 , 3.3
	T2, LOCF	22	10.3 (4.1)	8.82	7.7 , 12.7	4.7 , 20.7	22	-1.38 (1.62)	-0.55	-3.3 , 0	-4.5 , 0
	T3, LOCF	22	8.88 (2.94)	9.02	6.6 , 10.7	4.7 , 17.1	22	-2.8 (2.75)	-3.34	-3.7 , -1.1	-11.1 , 3.3
Control	T1	31	10.81 (2.2)	10.03	9.2 , 12.1	8.2 , 17.7					
	T2	26	10.16 (2.34)	10.65	8.5 , 11.8	4.4 , 14.4	26	-0.72 (2.86)	-0.64	-2.6 , 1.2	-5.8 , 4.9
	T3	23	10.54 (3.15)	11.27	7.9 , 12.4	4.9 , 18.8	23	-0.45 (3.47)	-0.66	-3.3 , 2.4	-6.4 , 6.7
	T2, LOCF	31	10.21 (2.26)	10.58	8.6 , 11.9	4.4 , 14.4	31	-0.6 (2.62)	-0.29	-2.6 , 0.9	-5.8 , 4.9
	T3, LOCF	31	10.15 (2.86)	9.58	7.9 , 11.8	4.9 , 18.8	31	-0.66 (3.1)	-0.71	-3.3 , 1.4	-6.4 , 6.7

The difference of the change from baseline after 12 weeks between the 2 groups was tested using the 2-sample t-test: P-value = 0.0402
The difference of the change from baseline after 12 weeks (LOCF) between the 2 groups was tested using the 2-sample t-test: P-value = 0.0125

*) T1 = Baseline ; T2 = Week 4 ; T3 = Week 12
